# Surface Mesoscopic Characterization and Analysis of Nanosecond-Laser-Processed Molybdenum for the Optimization of Antibacterial Performance

**DOI:** 10.3390/nano15040269

**Published:** 2025-02-11

**Authors:** Jie Wang, Qingyu Si, Jia Lv, Ruohan Chen, Qiuyu Sun, Yong Gao, Jing Zhang, Zhiming You, Sheng Wang, Bei Han

**Affiliations:** 1 Shaanxi Engineering Research Center of Advanced Nuclear Energy, Shaanxi Key Laboratory of Advanced Nuclear Energy and Technology, School of Nuclear Science and Technology, School of Energy and Power Engineering, Xi’an Jiaotong University, Xi’an 710049, China; wangjie1@xjtu.edu.cn (J.W.);; 2 XJTU-Huzhou Neutron Science Laboratory, Science Valley Medium-Sized Building #1, Huzhou 313000, China; 3 School of Public Health, Health Science Center, Xi’an Jiaotong University, Xi’an 710049, China

**Keywords:** molybdenum, surface, nanosecond laser

## Abstract

Molybdenum has gained attention as a promising biomedical material due to its excellent mechanical properties and inherent antimicrobial activity. However, challenges remain in developing facile fabrication methods and further enhancing its antimicrobial efficacy. This study pioneers the investigation of biofilm inhibition by laser-treated molybdenum sheets against *Pseudomonas aeruginosa* (ATCC27853) and *Staphylococcus aureus* (ATCC25923). The experimental results demonstrate that nanosecond-pulsed laser processing significantly suppresses biofilm formation, reducing the minimum optical density (OD) values by 25.3% and 64.9% for the two bacterial strains, respectively. The laser treatment modifies the surface morphology of molybdenum by eliminating defects, reducing the effective contact area, and lowering hydrophobicity. Additionally, localized laser heating induces the formation of MoO_3_ on the surface, which reacts with water to generate molybdic acid—a key contributor to antibacterial activity. These findings highlight nanosecond-pulsed laser processing as a cost-effective, scalable surface-modification strategy for medical-grade molybdenum. This approach holds significant potential for broadening the antimicrobial applications of molybdenum-based biomedical devices and implants.

## 1. Introduction

Metal materials are extensively utilized in the medical field, particularly in orthopedics, dentistry, and related disciplines, owing to their diverse range and robust mechanical properties. These materials are frequently employed to fabricate implants designed to replace damaged tissues or restore physiological functions. Given their critical role in medical devices, investigating the antimicrobial properties of metallic materials is essential for ensuring implant hygiene and patient safety. Antimicrobial properties in this context refer to the capability of a material to inhibit the growth of microorganisms. A key indicator of such properties is the inhibition of bacterial biofilm formation through the surface modifications of metals. Notably, environmental factors such as antibiotics, salts, and toxic heavy metals can promote biofilm tolerance [1]. It mediates adhesion between cells and the formation of biofilms, which envelope bacteria and protect them from the immune system [2].

Bacterial biofilm formation is part of the bacterial survival mechanism [3]. This can allow bacteria to evade host defenses and hide from antibiotics [4]. The process model of biofilm formation can be summarized by two steps. First, bacteria adhere rapidly to biomaterial surfaces through physicochemical interactions, including gravitational forces, van der Waals forces, electrostatic repulsion, and ion–dipole interactions. Second, adherent bacteria undergo proliferation and molecular-mediated intercellular aggregation, eventually forming multilayered clusters. Despite this general framework, the precise mechanistic details of biofilm formation remain unresolved. This uncertainty arises from the complex interplay of factors such as the dynamic in vivo environment, material surface properties, and bacterial adaptive behaviors, which collectively influence biofilm dynamics.

Molybdenum stands out as a notable metal in biomedical applications. As a key component of stainless steel and titanium alloys, its antibacterial properties have been extensively researched. Civa [5] found in his research on urban water pipes that the bacterial corrosion of 304 stainless steel pipe without molybdenum was much more serious than that of 316 stainless steel containing molybdenum. These pipes could release a small amount of molybdenum ions in flowing fresh water. Percival studied the outcome of molybdenum on the film formation of bacteria in a freshwater environment [6]. The findings revealed that molybdenum ions exerted significant inhibitory effects on bacterial biofilm formation. To systematically evaluate the antimicrobial behavior and surface characteristics of molybdenum, pure molybdenum sheets were employed in this study.

To further improve the antimicrobial properties of the molybdenum element, surface treatments also be applied to the hybrid nickel–molybdenum bimetallic sulfide nanozymes, including oxidation, enzyme immobilization techniques, and laser radiation [7]. Laser radiation used in the synthesis of nickel–molybdenum bimetallic sulfide contributes to a solution for the improvement of textural properties and increased exposed edge sites and sulfur vacancy. These result in higher haloperoxidase-like activity for L-NiMoS_2_ and better antibacterial ability.

Oxidation and enzyme immobilization techniques typically require the synthesis of specialized compounds to amplify a material’s antimicrobial efficacy. Unlike approaches involving the laser irradiation of solutions containing nickel–molybdenum bimetallic sulfide nanozymes, our method applies a nanosecond-pulsed laser treatment directly to the molybdenum surfaces. This localized thermal energy induces surface oxidation, generating molybdenum oxides (e.g., MoO_3_). Notably, under humid conditions, MoO_3_ undergoes hydrolysis to form hydronium ions (H_3_O⁺) and molybdate ions (MoO_4_^2−^)—both of which exhibit intrinsic antimicrobial activity. Hydronium ions (H_3_O^+^) reduce the solution pH, and hydrogen ions penetrate cell membranes to impede cell proliferation, which destroy their enzymes, transport systems, and DNA, as well as weaken bacteria adhesion [8,9,10]. Moreover, polymers and metals surfaces modified with MoO_3_ were exempt from microorganisms in after being in an infectious solution for 6 h [11]. The antibacterial properties of MoO_3_ are attributed to its oxidative stress properties and disruption of bacterial cell walls by its sharp edges [8]. It can be postulated that the generation of Mo oxides at the nanoscale level is the key to boosting antimicrobial performance.

Laser treatment is widely used due to its low cost and high treatment quality. It also has the ability to rapidly change the surface morphology of the sample to produce nanoscale periodic structures. M. Birnbaum first proposed laser-induced periodic surface structures (LIPSSs) [12]. Moreover, a LIPSS is produced through the interference of an incident laser beam with the material’s surface [13]. For pulsed laser systems, experiments have extensively observed that in addition to the irradiation wavelength and polarization direction, the laser flux and the number of pulses at the irradiation point are the keys to controlling the occurrence of LIPSSs. In the last two decades, LIPSS research has made great progress with the formation of typical ripples whose periods are smaller than the laser wavelength [14,15]. These structures are called high-spatial-frequency ripples (HSFLs), sometimes referred to as nano-arrays, and they must be clearly distinguished from classical near-wavelength size ripples (LSFLs).

The enhanced antibacterial efficacy was attributed to the microstructural modifications induced by laser surface treatment. Shazia Shaikh et al. [16] performed LIPSS treatment on Ti_6_Al_4_V and studied its antibacterial properties. It was found that after laser induction treatment, different forms of oxidized or semi-oxidized titanium dioxide microstructures were formed on the surface of the alloy, which reduced the wettability and improved the antibacterial performance. Gendy et al. [17] investigated the effect of silver nanoparticles ablated by a femtosecond laser on the growth kinetics of MRSA. Irradiation with a 400 nm laser was found to significantly reduce bacterial growth.

Some nanomaterials can be endowed with bactericidal properties according to their specific structure and composition, and often, they also act as carriers of antibiotics and natural antimicrobial compounds [18]. Mo-based nanomaterials kill bacteria or act as drug carriers, and they can even be used in combination with other nanomaterials to enhance the bactericidal effect with a photo/thermal response to heal wound infections [19]. However, molybdenum exhibits increased strength after appropriate heat treatment and is biocompatible with living organisms [20]. It can be used in small bioresorbable implants such as stents [21].

Consequently, laser treatment offers a straightforward procedure for achieving a surface nanostructure and regulating the optical, mechanical, and chemical properties of the molybdenum surface. The aim of this research is to modify the surface morphologies and chemical states of molybdenum by altering the laser treatment parameters, and subsequently assess the impact of laser treatment on the antimicrobial capabilities of molybdenum. Through the characterization of the surface morphology and chemical state of molybdenum, we can elucidate the connection between the surface properties and the bactericidal properties of laser-treated molybdenum. This will enable us to determine the parameter settings for laser treatments that optimize the antimicrobial properties.

## 2. Materials and Methods

### 2.1. Laser Parameters

The experimental sample comprised a 10 mm × 10 mm × 0.5 mm specification of pure metal molybdenum sheets, and the purity of the molybdenum was 99.95 at%. The specific laser processing parameters are shown in Table 1. The sample was treated by K20-CS (Han’s Laser, Shenzhen, China). For all the laser-treated Mo sheet samples, the parameters of laser pitch spacing, frequency, and wavelength were 20 μm, 20 kHz, and 1064 nm, respectively. And, the average power and laser spot were 6.67 W and 15 μm. The duration of each pulse was 200 ns. Moreover, samples #1, #2, and #3 were processed using a line hatched pattern. The spot size was set by a computer. The raster could be used to shape the laser beam by adjusting the grating’s period and intensity distribution so that the output was in a specific shape.

### 2.2. Biofilm Experiments

**Figure 1:**
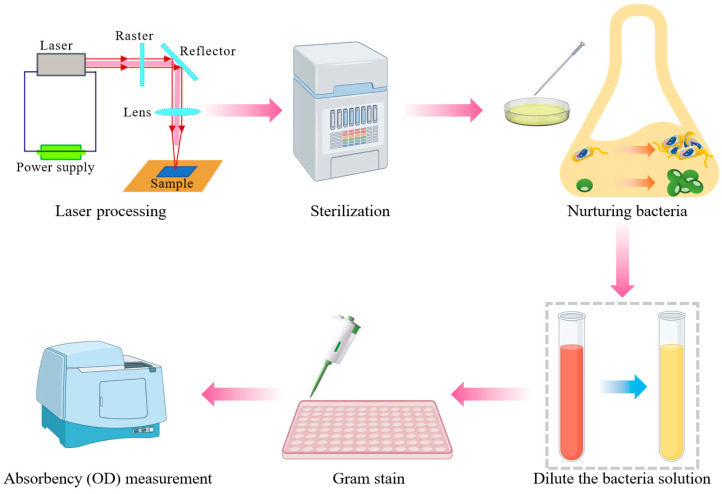
Biofilm experimental workflow.

The biofilm experimental workflow is briefly depicted in Figure 1. Pure molybdenum sheets with dimensions of 10 mm × 10 mm × 0.5 mm had their surfaces processed using a nanosecond laser. For the antibacterial property tests, the following materials were utilized: Biosharp BL601A (Hangzhou Mingte Biotechnology Co., LTD., Hangzhou, China) 0.01 M phosphate-buffered saline (PBS), 1% crystal violet, a 24-well plate with a pore size of 16 × 17 (h) mm, a 96-well plate with a pore size of 6.7 × 10.3 (h) mm, tryptone soybean broth (TSB) basal medium CM301, and anhydrous glucose.

*Staphylococcus aureus* was cultured in 30 g/1000 mL TSB medium supplied with 5% anhydrous glucose. Meanwhile, *Pseudomonas aeruginosa* was cultured in a 30 g/1000 mL TSB medium. The buffer was placed into a glass bottle, and the medium was transferred to an Erlenmeyer flask. Each Erlenmeyer flask was filled with 50–100 mL of the medium. After capping, the flask along with the sample was sterilized.

**Figure 2:**
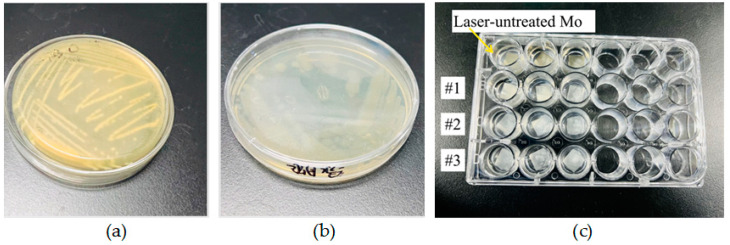
(**a**) *Staphylococcus aureus* ATCC25923; (**b**) *Pseudomonas aeruginosa* ATCC27853; (**c**) image of *Staphylococcus aureus* ATCC25923 biofilm on the surface of molybdenum sheet samples.

*Pseudomonas* spp. are widely distributed in food and processing environments, where they form biofilms that interact with other bacteria [22]. Therefore, in this experiment, *Staphylococcus aureus* (*S. Aureus*, Gram-positive bacteria) ATCC25923 and *Pseudomonas aeruginosa* (*P. aeruginosa*, Gram-negative bacteria) ATCC27853 were used, as shown in Figure 2. Aseptic operations were carried out on the ultra-clean workbench. The operation table was sterilized under ultraviolet light. Once the sterilization process was finished, the seed bacterial cells were added to the medium. Then, the medium with the added cells was incubated at 37 °C either in an incubator BG-80 (Shanghai Boxun Medical Biological Instrument Corp., Shanghai, China) or in an orbital shaker incubator SHA-C (XINBODE, Tianjin, China) operating at 200 revolutions per minute (rpm) for 24 h.

Once the bacteria were cultured, the bacterial solution was diluted with PBS buffer until its absorbance reached 1. The molybdenum (Mo) sheet samples were placed and numbered on 24-well plates, as illustrated in Figure 2c. After 5 min had passed, 1000 μL of the diluted bacterial solution was added drop by drop to each well in equal amounts. Subsequently, the 24-well plates were incubated at 37 °C in an incubator for 24 h. After that, the plates were processed using crystal violet staining, a test that is widely used in clinical microbiology laboratories [23,24].

During the crystal violet staining process, the following steps were carried out. First, 1 mL of the bacterial solution was aspirated. When aspirating the liquid, care was taken to draw it from the edge of the well plate to avoid touching the molybdenum sheet and thus preventing damage to the biofilm. The well was then washed three times with PBS buffer. Next, 1 mL of methanol was added to the well and left in place for 15 min. After this time, the methanol was aspirated. Subsequently, 1 mL of crystal violet was added and allowed to remain for 15 min before being aspirated. Following that, the well plate was rinsed three times with deionized water. Afterward, 1 mL of absolute ethanol was added and left for 15 min. Finally, the liquid in each well was divided into three approximately 200 μL portions. These portions were transferred to a 96-well plate for absorbance measurement. The formation of the biofilm was characterized by using a visible spectrophotometer 722N (INESA Electronics Co., Ltd., Shanghai, China) to measure the absorbance of the bacterial solution. A smaller absorbance indicated a lower concentration of the bacterial solution, suggesting that the conditions were less favorable for biofilm formation.

**Figure 3:**
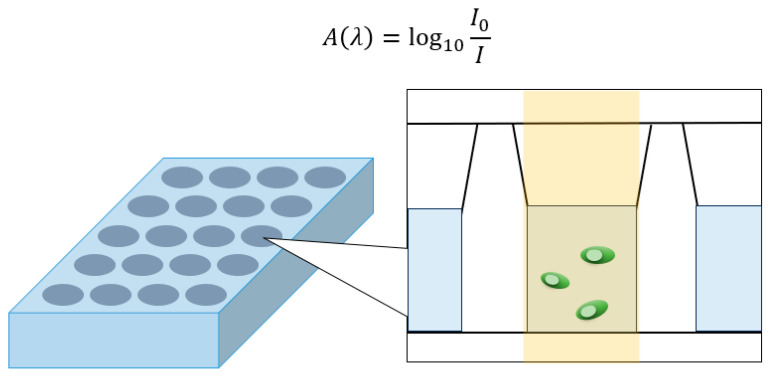
The principle of OD measurement of antibacterial performance.

The term “absorbance” is defined as the capacity of a solution to absorb light of a particular wavelength, which is also known as the optical density (OD). The specific calculation formula [25] and principle of OD are shown in Figure 3. In this experiment, the antibacterial performance is primarily determined by washing the biofilm off the metal sample and then measuring the concentration of the bacterial solution using a microplate spectrophotometer. This measurement serves as the evaluation criterion for the antibacterial performance. In this paper, the OD is measured at a light wavelength of 595 nm. Here, *I* represents the intensity of light at the specified wavelength *λ* after it has passed through the sample (transmitted light intensity), and the ratio *I*₀/*I* represents the intensity of the light before it enters the sample. Additionally, microscopic characterization of the samples was carried out to explore the factors influencing the antibacterial properties.

### 2.3. Characterization Method

The surface morphologies of the molybdenum (Mo) sheets, both before and after laser processing, were examined using a scanning electron microscope (SEM, JEOL 7800F, Tokyo, Japan). The surface roughness of the samples was characterized by a laser scanning confocal microscope, the Leica DCM8 (LSCM, Leica Ltd., Solms, Germany), over a characterization area of 250 μm × 250 μm. For the spectroscopic analysis, spectra of Mo 3d, C 1s, and O 1s, and survey spectra were recorded using an Al Kα excitation source (Thermo Fisher ESCALAB Xi+, Thermo Fisher, Waltham, MA, USA). This was carried out under a pressure of less than 7 × 10^−9^ mbar. The binding energies were calibrated with reference to C 1s at 284.8 eV. The crystal structure of the Mo sheets before and after laser processing was evaluated through X-ray Diffraction (XRD, Bruker, Karlsruhe, Germany). The surface wettability was measured by a DSA100 Optical (KRUSS, Hamburg, Germany) contact-angle-measuring instrument. Moreover, the UV spectra of the sample were measured using a UV spectrophotometer, namely the Lambda 950 (PerkinElmer, Hopkinton, MA, USA).

## 3. Results and Discussion

**Figure 4:**
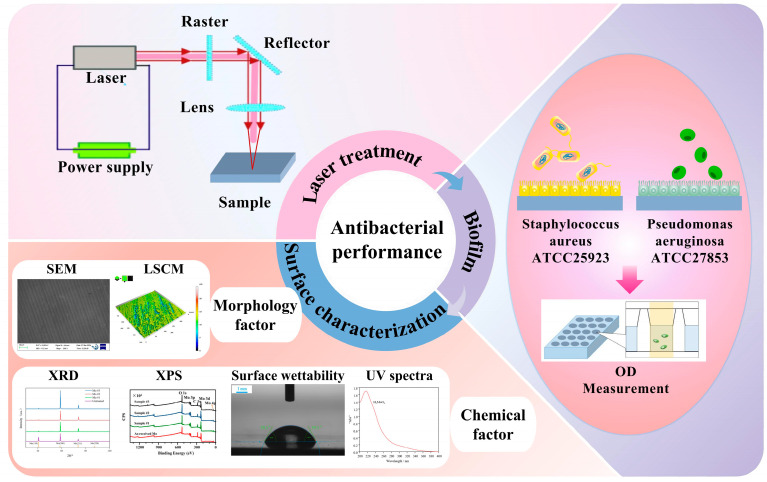
The experimental arrangement of this study.

To delve deeper into the specific details of how laser treatment impacts the antimicrobial properties of molybdenum, the experimental procedure is presented in Figure 4. A series of relevant characterizations were employed. These aimed to evaluate the factors contributing to the improvement of antimicrobial properties due to laser treatment, considering both the surface morphology and the surface composition of the samples.

In this paper, for the first time, laser treatment was applied to molybdenum wafers to systematically explore the impact of such treatment on their antimicrobial properties. SEM, LSCM, XRD, and XPS tests were carried out. These tests aimed to understand the causes of antimicrobial resistance by examining both the surface morphology and the surface composition of the samples.

In Section 3.1, the antibacterial properties of samples processed with different laser parameters were tested against *Staphylococcus aureus* ATCC25923 and *Pseudomonas aeruginosa* ATCC27853. Subsequently, in Section 3.2 and Section 3.3, the surface morphologies and surface roughness were analyzed to evaluate how laser parameters affected the surface topography of the Mo samples. Moving on to Section 3.4, the contact angle of the samples was studied because surface wettability can influence the growth of bacterial strains. Additionally, in Section 3.5, Section 3.6 and Section 3.7, the surface chemical states of the samples were analyzed using XRD, XPS, and UV spectroscopy. This was carried out to clarify the crystal structure, surface valence states, and the formation and presence of HMoO_3_ following laser processing.

### 3.1. Biofilm 

**Figure 5:**
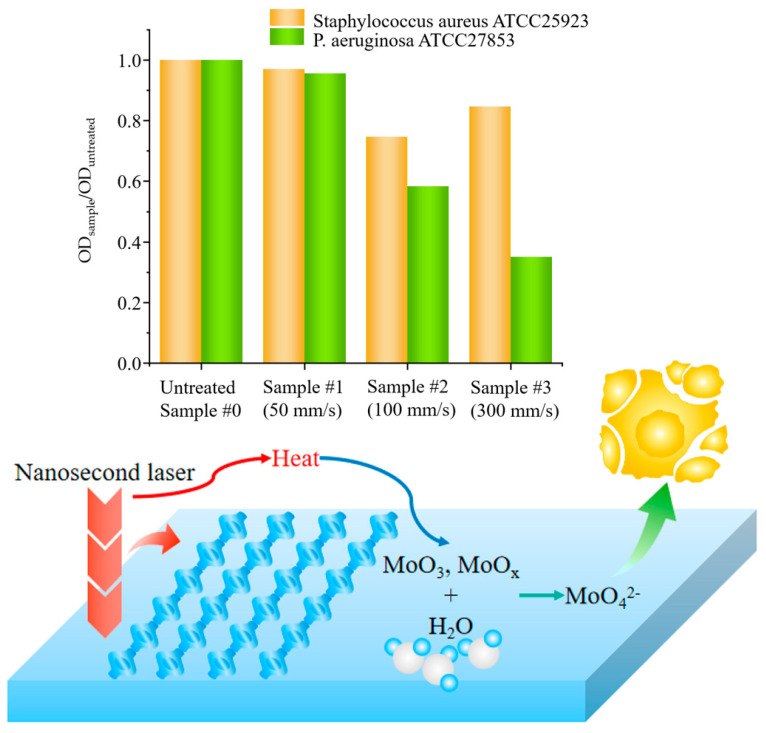
Comparison of biofilm growth of *Staphylococcus aureus* ATCC25923 and *Pseudomonas aeruginosa* ATCC27853 at different laser scanning rates.

After staining and washing the plates by crystal violet staining, the optical density (OD) of Mo samples with different laser processing parameters was measured. The ratios of absorbance from laser-treated Mo and that of untreated Mo (OD_sample_/OD_untreated_) were compared for *Staphylococcus aureus* ATCC25923 and *P. aeruginosa* ATCC27853, as shown in Table 2 and Table 3. Comparison of biofilm growth of *Staphylococcus aureus* ATCC25923 and *Pseudomonas aeruginosa* ATCC27853 at different laser scanning rates is shown in Figure 5.

The antibacterial results for *Staphylococcus aureus* ATCC25923 of laser-processed and unprocessed Mo sheets in Table 2 indicate that the antibacterial effects increased with the increase in laser scanning speeds and then decreased with the further slight increase in laser scanning speeds. The inhibitory mechanism of laser treatment and comparison of antimicrobial effects are shown in Figure 5.

The antibacterial results for *Pseudomonas aeruginosa* ATCC27853 on the laser-processed molybdenum sheets, presented in Table 3, demonstrate that laser processing can influence the antibacterial performance of the laser-treated Mo sheets. When the Mo sheets are laser processed at different scanning rates while keeping certain laser parameters constant, the antibacterial effect of the samples gradually improves. The antibacterial performance depends not only on the intrinsic properties of the bacteria but also on the surface morphologies and chemical states of the substrates.

When comparing molybdenum metal with and without laser treatment, the results indicate that the laser treatment method is advantageous. This treatment significantly enhances the antibacterial performance of the molybdenum metal sheet. The antibacterial composition and physical structure are key factors influencing the antibacterial properties of materials. According to the scanning electron microscopy results, as the laser scanning speed increases, the surface defects of the samples disappear. This reduces the effective surface area of the samples in contact with bacteria. As a result, as the laser scanning speed rises, the laser-treated molybdenum becomes less suitable for biofilm formation.

Furthermore, laser treatment causes an increase in the molybdenum oxide content of the sample. This enhances the hydrophilicity of the sample material, leading to the reaction of MoO_3_ with water to form H_2_MoO_4_, which inhibits bacterial growth.

### 3.2. SEM

**Figure 6:**
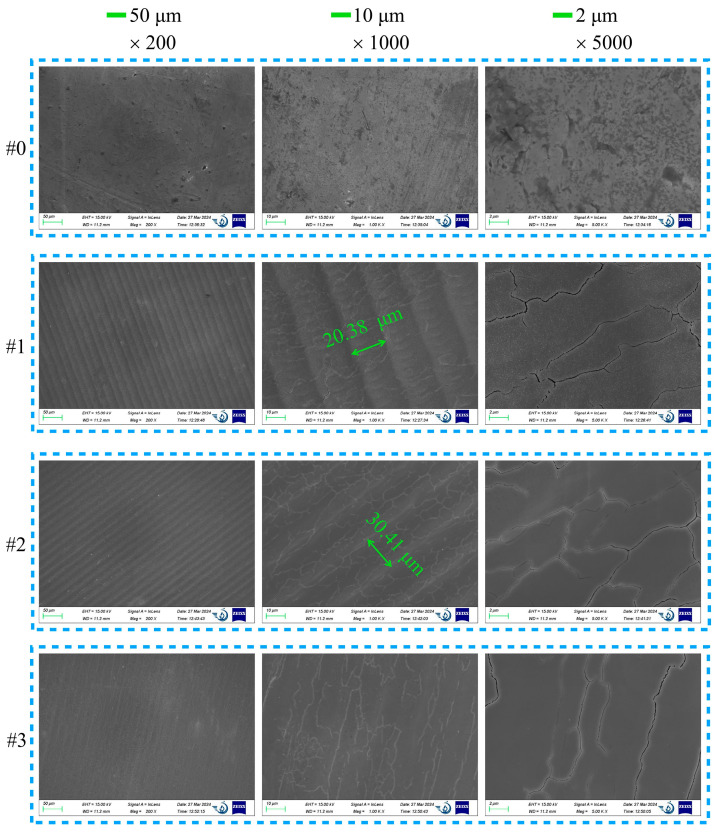
SEM images of as-received Mo samples #0 and laser-processed Mo samples #1, #2, and #3.

Surface morphology tests were conducted to examine the periodic structure on the molybdenum surface generated by laser treatment, as well as the removal of defective scratches on the sample’s surface. Figure 6 presents SEM images of the as-received Mo sample #0, along with laser-processed Mo samples #1, #2, and #3, captured at different magnifications.

**Figure 7:**
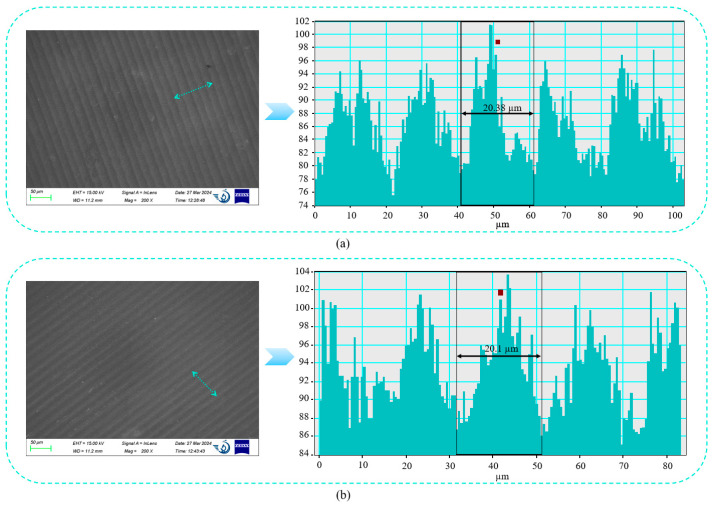
Two-dimensional FFT map of the corresponding SEM image. (**a**) 2D-FFT map sample #1; (**b**) 2D-FFT map sample #2. The FFT map shows the information along the bule arrows region.

Based on the 2D-FFT map of the corresponding SEM image in Figure 7, the spacing dimensions of the periodic structures on the sample surface can be precisely determined. When the laser scanning rate is 50 mm·s^−1^, the surface of sample #1 exhibits regular periodic fringes with a spacing of approximately 20.38 μm, compared to the as-received surface, which aligns with the laser parameter setting. Through comparing the magnifications of ×1000 and ×5000, it was observed that after laser treatment, the defects and scratches on the original surface were diminished, and the cracks became denser and finer. The spacing size of sample #2 was 20.1 μm, which is close to 20 μm. This indicates that the spacing of the LIPSSs (laser-induced periodic surface structures) is near the pitch spacing (20 μm) of the laser. From the ×1000 magnification images of samples #1, #2, and #3, as the laser scanning rate increases, the periodic fringes on the surface become less distinct, and the number of fine cracks on the surface reduces. As the scanning speed rises, the original surface of the molybdenum sample becomes smoother.

References [26,27] reported that as the period of low-spatial-frequency LIPSSs (LSFLs) is usually very close to the irradiation wavelength, the structures produced by laser treatment in this paper were determined to be LSFLs. When the laser scanning speed is 50 mm·s^−1^, the laser power is sufficiently high to eliminate the surface defects of the original sample. Nevertheless, distinct laser scanning traces are still present. As the scanning rate increases, the sample surface gradually becomes flatter, and the number of fine cracks also diminishes. This indicates that laser treatment effectively polishes the molybdenum surface.

### 3.3. LSCM

**Figure 8:**
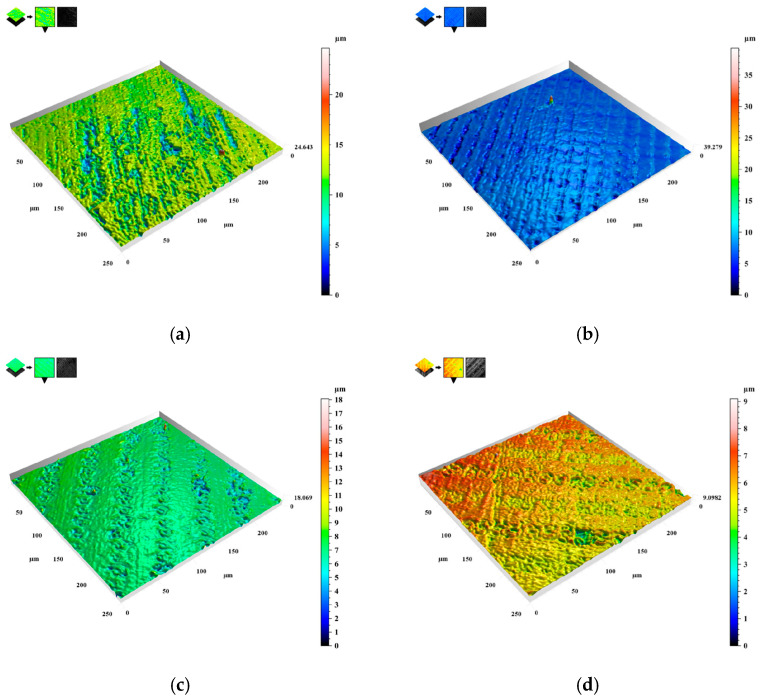
Three-dimensional image of sample surface roughness; (**a**) laser untreated sample, (**b**) sample #1, (**c**) sample #2, and (**d**) sample #3.

Based on the SEM results, it is depicted that the laser treatment led to the elimination of defective scratches on the surface of the samples, which can be more intuitively obtained from the roughness results. The 3D characterization images of the surface roughness of samples #0–#3 are shown in Figure 8. *Sa* was introduced as the main parameter for sample roughness assessment according to ISO 25178 [28,29]. As is depicted in Figure 8a, the surface of the laser-untreated sample is marred by more scratches, with a roughness parameter *Sa* of 1.22 μm. In contrast, the surface of the laser-treated sample exhibits distinct periodic structures, corroborating the SEM findings. As the laser scanning rate increases, the sample surface progressively becomes flatter. The *Sa* values of sample #1, sample #2, and sample #3 are 0.53 μm, 0.48 μm, and 0.42 μm, respectively. This shows that an increase in the laser scanning rate leads to a reduction in the surface roughness of the samples. Consequently, the effective contact area between the bacteria and the sample surface decreases.

### 3.4. Surface Wettability

**Figure 9:**
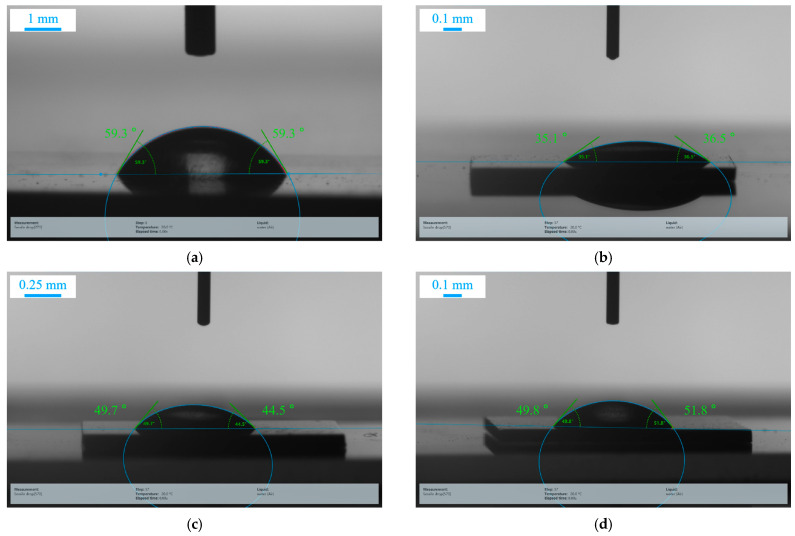
Images of water contact angle tested on (**a**) laser untreated sample, (**b**) sample #1, (**c**) sample #2, and (**d**) sample #3.

The water-contact-angle test results of the untreated Mo sample #0, and laser-treated samples #1, #2, and #3 are shown in Figure 9. The untreated Mo surface was hydrophilic, having a contact angle of 59.3 ± 0.5°. The wettability of the laser-treated samples #1, #2, and #3 with water indicate that the Mo surface turned superhydrophilic, with a contact angle of 35.1–36.5°, 44.5–49.7°, and 49.8–51.8°, respectively. This suggests that a biofilm might grow relatively easily on the surface of laser-processed Mo with a low laser scanning speed due to the low contact angle. The antibacterial property of the laser-processed Mo surface may be related to the formation of molybdate. Additionally, laser treatment in air can increase the quantity of molybdenum oxides formed, which could lead to a corresponding increase in the formation of molybdate.

It can be indicated that the laser treatment resulted in a decrease in the contact angle of molybdenum, thus inhibiting the formation of a biofilm. This is because laser treatment can effectively minimize the defects on the molybdenum surface. However, as the laser scanning rate rises, the sample’s contact angle gradually increases. This suggests that the enhanced hydrophilicity of the sample promotes the formation of molybdate, which is associated with the sample’s antimicrobial properties. Moreover, the antimicrobial properties of a material do not solely rely on the contact angle size. Instead, they depend on the synergistic influence of factors such as the material’s surface morphology and composition. Consequently, these aspects should be considered holistically.

### 3.5. XRD

In addition to causing a change in the surface morphology of the sample, the heat of the laser treatment could also change the crystal orientation of that sample. Thus, the surface crystal properties were studied in this section.

**Figure 10:**
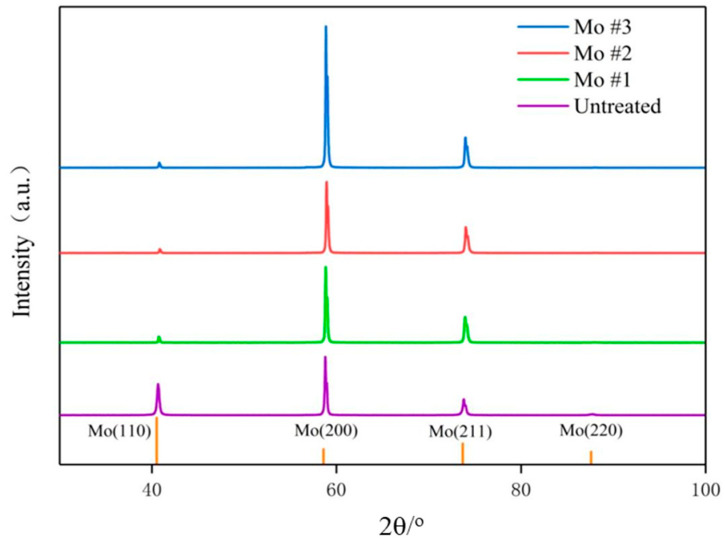
XRD results of as-received Mo sample #0, and laser-processed Mo samples #1, #2, and #3.

Figure 10 shows the XRD results of sample Mo #1, #2, and #3, and untreated Mo sheets. It can be indicated that samples Mo #1, #2, and #3, and untreated Mo sheets are matched to Mo(110), Mo(200), and Mo(211) at the measured peaks of diffraction intensity angles of 40.51°, 58.61°, and 73.68°, respectively. For the untreated Mo sheets, there was also a faint peak at 87.595° matched to Mo(220). With the increase in laser processing scanning speed, the diffraction intensity peaks at 40.51° and 87.60° weaken, and the diffraction intensity peaks at 58.61°and 73.68° increase. This shows that the laser-treated sample of Mo mainly exists in the form of crystals with three crystal indices: Mo(110), Mo(200), and Mo(211). The crystal grain sizes of samples #0, #1, #2, and #3 are 30.5 nm, 132.5 nm, 57.8 nm, and 42.6 nm, respectively. It can be found that laser treatment increases the grain size of the sample, and it decreases as the laser scanning rate increases. From this result, we can examine the surface composition of different samples before the formation of the biofilm and the crystallinity of the surface composition after laser treatment.

On the basis of the XRD results, we can examine the surface crystalline phase as well as the grain size of different samples before the biofilm formation. According to the study of M. Naghizadeh et al. [30], it can be indicated that by decreasing the grain size, the yield strength and tensile strength of the metal are increased, which shows that the laser treatment can effectively enhance the mechanical properties of molybdenum.

### 3.6. XPS

As mentioned above, the antibacterial or bactericidal performance of laser-treated Mo is relevant to the formation of H_2_MoO_4_, which is formed by the reaction of molybdenum oxides on the surface of the sample with water, leading to deteriorating cell growth and proliferation [31,32] and a significant reduction in its adhesion [11]. Therefore, an interpretation of the Mo 3d scan XPS spectra of the as-received Mo plate and laser-treated samples with different laser processing parameters was performed. The surface oxidation states of Mo plates before and after laser processing were analyzed using the results of the XPS investigation and a survey of the published literature. This indicates that four distinct molybdenum states (Mo metal, Mo (4+), Mo (5+), and Mo (6+)) need to be taken into account. By fitting the Mo 3d signal shapes in the spectra of the Mo plate samples treated with different laser parameters, estimates can be made for the amounts of Mo metal, Mo (4+), Mo (5+), and Mo (6+). The Mo 3d and O 1s signal shapes are fitted using CasaXPS 2.3.18, with line shapes of LF(1, 1, 55, 260) and GL(30), respectively.

**Figure 11:**
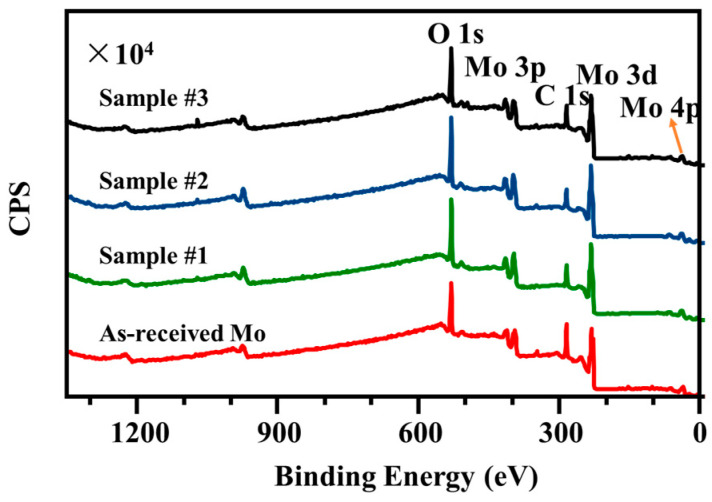
XPS wide-scan spectra of as-received Mo, and laser-treated Mo sample #1, sample #2, and sample #3.

Figure 11 presents XPS wide-scan spectra of the as-received Mo, and laser-treated Mo sample #1, sample #2, and sample #3. The qualitative XPS wide-scan analyses of the sample surface implies the presence of O, Mo, and C, which were the main elements on the laser-treated and untreated Mo samples. The most evident difference between the surfaces of these four Mo samples (as-received Mo, and laser-treated Mo sample #1, sample #2, and sample #3) appears in the carbon, Mo, and oxygen peaks.

The decrease in the carbon peak intensity in Figure 11 indicates that the carbon contamination on the as-received Mo sheet sample surface was partially removed upon laser processing in air. One also observes the increase by 2.5–10.3% in the oxygen peak height and the decrease by 18.2–26.0% in the carbon peak. This variation is generally recognized to be a direct consequence of the surface oxidation of the Mo sheets after laser processing in air. Thus the Mo/O ratios decreased for the laser-treated samples #1, #2, and #3 by 56.7%, 59.1% and 61.1%, respectively, compared with that of as-received case of 62.7%.

**Figure 12:**
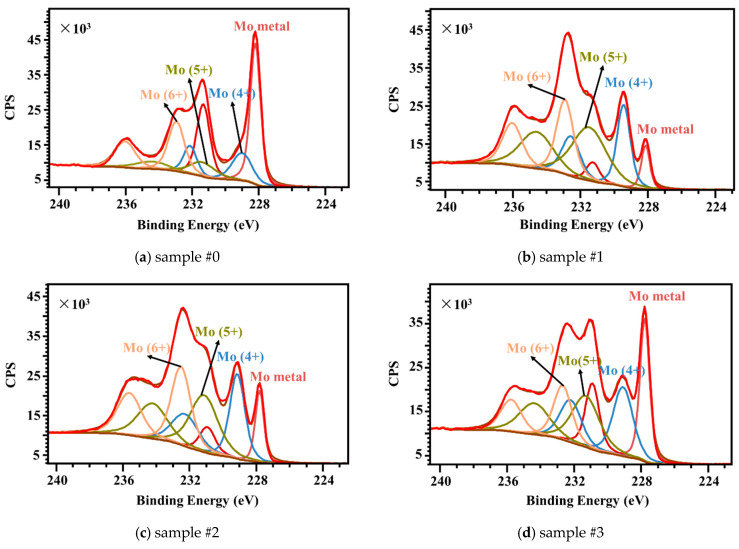
Fitted Mo 3d XPS spectra of (**a**) as-received Mo, and laser-treated Mo (**b**) sample #1, (**c**) sample #2, and (**d**) sample #3.

Figure 12a–d show the Mo 3d XPS core-level spectra of the as-received Mo, and laser-treated Mo sample #1, sample #2, and sample #3. The Mo 3d spectra display a characteristic of 3d 5/2 and 3d 3/2 induced by the spin–orbit coupling. The line shape of the doublet, Mo 3d 5/2, and Mo 3d 3/2 is synthetic Lorentzian asymmetric (LF) LF(1, 1, 55, 260), which was selected based on the Lorentzian energy distribution of electrons and the Gaussian instrumental broadening. The spin–orbit splitting and binding energy of Mo 3d are in accordance with the values reported by other researchers [33,34]. The full width at half maximum (FWHM) corresponding to the Mo 3d line was calculated and the line of the as-received Mo sample was narrower than those of the laser-treated samples. The narrowing is supposed to be caused by the electrical environment and the mixed valence states of Mo metal and Mo oxides. The deconvolution of the Mo 3d peaks reveals four components that correspond to different Mo valence states. The component peaks at 232.5–232.9 eV, 231.2–231.6 eV, 229.0–229.4 eV, and 227.7–228.1 eV are expected to be Mo (6+), Mo (5+), Mo (4+), and Mo metal. After laser processing in air, the peaks of Mo 3d lines become broader and less resolved, manifesting a reduction in the Mo surface crystallinity. The XPS spectrum reveals the concentrations of Mo metal and Mo oxides changing, which is probably the effect of the inward diffusion of oxygen on the Mo sheet surface after laser processing.

Table 4 shows that the concentrations of Mo metal increased with the increase in laser scanning speed, which were 8.3 at%, 12.3 at%, and 26.9 at%, respectively, compared to the untreated sample at 45.8 at%. The Mo oxide concentration of sample #1 is the highest in Table 2, which demonstrates that the production of H_2_MoO_4_ produced by the conversion of molybdenum oxides with bacteriostatic properties is relatively high [35]. Nonetheless, the antibacterial performance can also be influenced by the surface morphologies and surface wettability.

**Figure 13:**
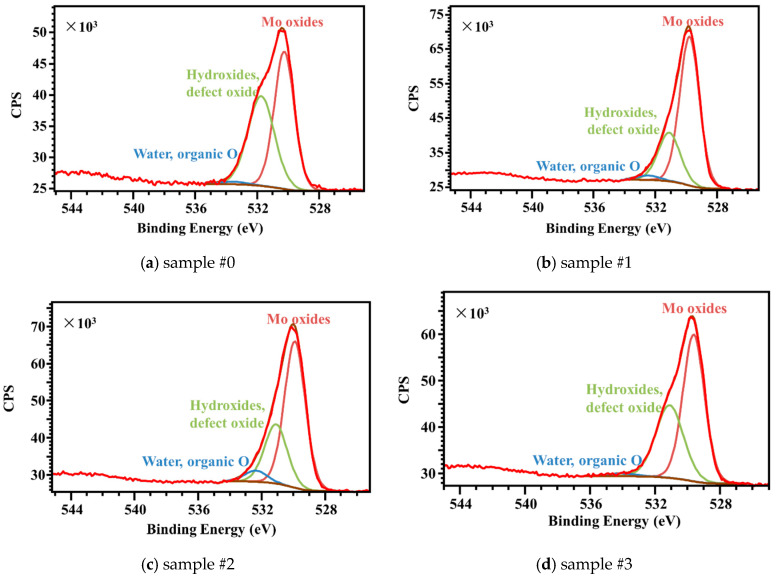
Fitted O 1s XPS spectra of (**a**) as-received Mo, and laser-treated Mo (**b**) sample #1, (**c**) sample #2, and (**d**) sample #3.

Figure 13 shows the characteristic core-level peaks for the O 1s state of as-received Mo, and laser-treated Mo samples #1, #2, and #3. The O 1s binding energies of Mo oxides were found to be 530.3–530.5 eV, which are in agreement with the reported values stated in references [36,37,38,39,40]. The concentrations of Mo oxides for samples #0, #1, #2, and #3 were evaluated as 52.1%, 58.4%, 61.5%, and 69.8%, respectively. While, the hydroxides and defect oxides contents were 46.4%, 34.3%, 30.9%, and 27.9% for these four samples, respectively. Moreover, the water and organic O concentrations were less than 7.4% for all the tested samples.

### 3.7. UV Spectra of MoO_3_ Infiltrated in Water

**Figure 14:**
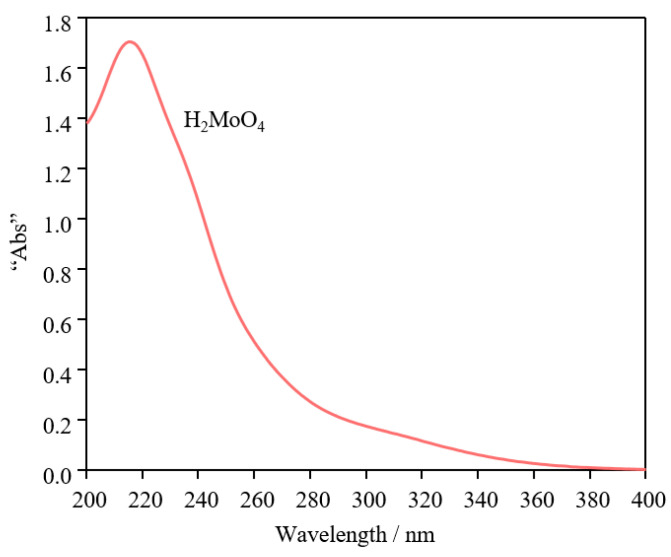
The UV spectra of the solution obtained from laser-treated molybdenum infiltrated in water.

In order to verify the specific chemical components that inhibit the growth of biofilms on the laser-treated molybdenum surface, the UV spectra of the solution obtained from laser-treated molybdenum infiltrated in water is shown in Figure 14. The XPS results explained that the laser treatment produces MoO_3_, and that MoO_3_ reacts with water in the wet environment to produce H_2_MoO_4_ [11]. The spectral peak in Figure 13 matches with H_2_MoO_4_ [41], which shows that the specific antimicrobial composition of the laser-treated molybdenum surface is H_2_MoO_4_. The equation explains the reaction of the molybdenum oxide surface with water, forming H_2_MoO_4_, and the graphs’ x-axes show the wavelength while the y-axes represent the “abs”. In addition, the laser treatment led to an increase in the hydrophilicity of the molybdenum surface, which promoted the formation of H_2_MoO_4_, leading to a further increase in the antimicrobial performance of the laser-treated molybdenum.


(1) $$\begin{array}{c} {\mathrm{MoO}}_{3}+H_{2}O⇌H_{2}{MoO}_{4} \end{array}$$

## 4. Conclusions

In this paper, Mo sheets were treated with different laser parameters and biofilm experiments were carried out and studied initially. It was found that laser processing had a positive impact on the antibacterial properties of Mo sheets.

(1) The laser-treated molybdenum sheets significantly inhibited the biofilm formation of both *Staphylococcus aureus* ATCC25923 and *Pseudomonas aeruginosa* ATCC27853. Their minimum OD values decreased by 25.3% and 64.9%, respectively, when compared with the untreated laser samples. Additionally, as the laser scanning speed increased, the roughness of the sample surface decreased. This reduction in roughness led to a decrease in the effective contact area between the bacteria and the sample surface, which was detrimental to bacterial growth.
(2) The antimicrobial performance of untreated and laser-treated molybdenum flakes was related to the surface chemical state, surface morphology, and inherent characteristics of bacteria. Laser treatment can effectively change the surface morphology and oxygen content of the sample. Specifically, MoO_3_ reacts with water to generate H_2_MoO_4_, inhibiting the formation of the bacterial biofilm.
(3) Laser treatment also modified the surface crystallinity of metallic molybdenum. As the scanning rate increased, the grain size gradually decreased. This decrease in grain size was beneficial for enhancing the mechanical strength of the material. The improved mechanical properties of molybdenum contributed to its corrosion resistance.

The characterization results indicate that the modification of the chemical composition, surface morphology, and the effective contact area with bacteria have a more substantial impact on the antimicrobial properties than factors such as the crystal structure and contact angle. Laser treatment offers the advantages of flexibility in regulating multiple parameters, lower cost, higher efficiency, and ease of manufacture for the rapid preparation of nanomaterials. Consequently, this study provides novel perspectives that pave the way for future research directions in this area. The findings highlight potential paths for further exploration in the pre-treatment and processing methods of emerging medical materials.

## Figures and Tables

**Table 1:** Laser parameters for processing the molybdenum samples.

Sample	Hatched Pattern	Pitch Spacing /μm	Scanning Speed /mm∙s^−1^	Average Power/W	Spot/μm
#0	As-received untreated Mo	/	/	/	/
#1	Line	20	50	6.67	15
#2	Line	20	100	6.67	15
#3	Line	20	300	6.67	15

**Table 2:** Comparison of antibacterial effects of Mo sheets after laser processing against *Staphylococcus aureus* ATCC25923.

Sample	OD_sample_ Test 1	OD_sample_ Test 2	OD_sample_ Test 3	Average OD_sample_	OD_sample_/OD_untreated_
#1	1.307	1.205	1.091	1.201	0.970
#2	0.979	0.821	0.975	0.925	0.747
#3	1.047	1.007	1.089	1.048	0.846
#0	1.073	1.372	1.268	1.238	1.000

**Table 3:** Comparison of antibacterial effects of Mo sheets after laser processing against *P. aeruginosa* ATCC27853.

Sample	OD_sample_ Test 1	OD_sample_ Test 2	OD_sample_ Test 3	Average OD_sample_	OD_sample_/OD_untreated_
#1	0.899	1.098	0.703	0.900	0.955
#2	0.590	0.546	0.511	0.549	0.583
#3	0.501	0.226	0.264	0.330	0.351
#0	0.965	0.964	0.897	0.942	1.000

**Table 4:** XPS curve-fitting analysis results of the concentrations of Mo metal and Mo oxides.

Sample	Mo Metal/at%	Mo (4+)/at%	Mo (5+)/at%	Mo (6+)/at%
#0	45.8	17.5	11.5	25.2
#1	8.3	23.8	40.0	27.9
#2	12.3	25.6	31.6	30.5
#3	26.9	26.4	27.4	19.3

## Data Availability

Data are available upon written request to the corresponding author.
